# Nutritional value and antioxidant capacity of organic and conventional vegetables of the genus *Allium*

**DOI:** 10.1038/s41598-022-23497-y

**Published:** 2022-11-04

**Authors:** Anna Czech, Marek Szmigielski, Iwona Sembratowicz

**Affiliations:** 1 Department of Biochemistry and Toxicology, Faculty of Biology, Animal Sciences and Bioeconomy, University of Life Sciences in Lublin, Akademicka 13, 20-950 Lublin, Poland; 2 Department of Biological Bases of Food and Feed Technology, University of Life Sciences in Lublin, ul. Głęboka 28, 20-612 Lublin, Poland

**Keywords:** Plant sciences, Nutrition

## Abstract

There are indications that organically grown plants are safer for health and have higher antioxidant content than conventional ones. Vegetables of the genus *Allium* L. are a valuable source of health-promoting substances, including compounds with antioxidant properties. The aim of the study was to compare the antioxidant potential and nutritional value of four species of *Allium* L. vegetables obtained from organic and conventional production: garlic, leek and red and yellow onion. Their proximate and mineral composition were determined, as well as the content of bioactive substances and antioxidant potential. The study showed that the cultivation method significantly influenced the parameters tested. Comparison of organic vegetables with conventional ones in terms of content of dry matter, crude protein and crude fibre revealed no general trend indicating the superiority of one method over the other. However, all organic vegetables analysed were more abundant in minerals (Ca, Mg, Fe, Zn, Cu and Mn) and bioactive compounds. They also exhibited higher antioxidant capacity as measured by the FRAP and DPPH^.^ tests. Their consumption (especially organic garlic and leek) may therefore strengthen the body's natural antioxidant defences and is beneficial for health.

## Introduction

Recent years have seen growing demand for high-quality, healthy food products containing valuable substances that can prevent various illnesses. Vegetables of the genus *Allium* L. are particularly worth noting as a source of antioxidants and anti-carcinogens^1,2^, as well as antibiotic compounds^2,3^ and substances exerting a beneficial effect on the cardiovascular system^4^. Unfortunately, these vegetables can also contain chemical pollutants in the form as pesticide residues and nitrates. For this reason, organic agricultural products, perceived as healthier and free of this type of contamination, are becoming increasingly popular^5^.

According to the principles of organic crop cultivation, easily soluble mineral fertilizers and chemical pesticides are not used. This may affect the levels of basic nutrients, mineral elements, and biologically active substances, which can differ considerably from those found in conventional products^6,7^.

The question of nitrogen availability for plants seems crucial in terms of synthesis of secondary metabolites such as polyphenols, carotenoids and vitamins. When easily available nitrogen from mineral fertilizers is present, the metabolism of plants is directed at production of nitrogen-containing compounds and growth processes, while the production of secondary metabolites based on a carbon skeleton is limited^8^. Increased synthesis of these compounds is the plant’s reaction to stress factors, as a kind of defence mechanism. Therefore organic vegetables, more vulnerable to attack by pests and pathogens, can be expected to contain higher levels of these valuable substances^7,9^. They may also have higher antioxidant potential, as many of these compounds function as antioxidants. Numerous studies comparing various products grown organically and conventionally appear to confirm the advantage of organic crops in terms of content of secondary metabolites and antioxidant properties^10–12^. There is no conclusive evidence, however, that organic products are of higher nutritional value or that consumption of them has greater health benefits.

Therefore the aim of the study was to analyse the effect of the cultivation method (organic vs. conventional) on the nutritional value and antioxidant capacity of vegetables of the genus *Allium* L. grown in Poland. An additional objective of the study was to compare the parameters tested between various *Allium* vegetables.

## Results and discussion

### Effect of cultivation system on proximate chemical composition

Analysis of the effect of the cultivation model on the dry matter content of the vegetables showed that conventionally grown garlic and leek contained significantly (p < 0.001) more dry matter than their organic counterparts (Tables 1 and 5). This is in contrast to the frequently observed tendency of higher dry matter content in organic products than in conventional ones^7,13,14^. Data gathered by Lairon et al.^15^ suggest that significant differences in dry matter content in favour of organic products are found in the case of root, leaf and tuberous vegetables. Apart from environmental factors, such as weather conditions during the growing season or soil type, the water content in vegetables may be influenced by fertilization. The large amounts of mineral nitrogen used in conventional production are conducive to vegetative growth, which is linked to an increase in the amount of water in vegetables, and thus a decrease in dry matter^8,16^. Irrespective of the cultivation system, the dry matter content was by far the highest in garlic and the lowest in red and yellow onion (p ≤ 0.01).

**Table 1:** Content of nutrients (g 100 g^−1^) in *Allium* vegetables depending on the cultivation model (conventional—C; organic—O).

Vegetable	Cultivation model	Garlic	Yellow onion	Red onion	Leek	Statistical analysis		
						VS	CM	VSxCM
Dry matter	C	34.63^a^ ± 2.04	11.12^c^ ± 0.96	10.43^c^ ± 1.19	23.36^b^ ± 3.01	**	ns	**
	O	28.82^a^ ± 2.72	10.66^c^ ± 0.73	10.21^c^ ± 0.83	17.11^b^ ± 1.79			
	Mean	31.73^a^ ± 3.79	10.90^c^ ± 0.773	10.33^c^ ± 1.01	20.24^b^ ± 4.09			
Crude protein	C	17.14^a^ ± 0.634	7.82^c^ ± 0.227	9.88^b^ ± 0.347	7.96^c^ ± 0.205	**	ns	**
	O	15.03^a^ ± 0.929	11.13^c^ ± 0.884	13.61^b^ ± 1.06	5.08^d^ ± 0.151			
	Mean	16.11^a^ ± 1.33	9.48^c^ ± 0.883	11.75^b^ ± 2.06	6.18^d^ ± 1.27			
Crude ash	C	2.32 ± 0.072	2.09 ± 0.024	2.30 ± 0.030	2.08 ± 0.352	ns	ns	ns
	O	2.12 ± 0.214	2.21 ± 0.065	2.12 ± 0.110	2.17 ± 0.305			
	Mean	2.22 ± 0.185	2.15 ± 0.066	2.21 ± 0.121	2.12 ± 0.321			
Crude fibre	C	1.63^c^ ± 0.061	4.17^a^ ± 0.178	4.05^a^ ± 0.213	3.27^b^ ± 0.202	**	ns	**
	O	0.87^c^ ± 0.049	4.16^a^ ± 0.037	3.57^b^ ± 0.136	3.53^b^ ± 0.132			
	Mean	1.25^c^ ± 0.397	4.16^a^ ± 0.037	3.81^ab^ ± 0.301	3.40^b^ ± 0.211			

*VS* vegetable species, *CM *cultivation model (conventional vs. organic), *ns *no statistics.

*Correlation significant at p ≤ 0.05 (one-way analysis).

**Correlation significant at p ≤ 0.01 (one-way analysis).

^a,b^^,c^Values with different superscript letters in a row are significantly different at p ≤ 0.05.

In addition to its influence on water content, the cultivation model can also affect the amount of protein in vegetables. While it is true that organic crops may contain less protein, according to some researchers^17,18^ the protein is of better quality, with a more favourable amino acid composition, i.e. higher content of some essential amino acids, such as lysine. Maggio et al.^19^, in an analysis of the amino acid composition of organic and conventional potatoes, found that the organic ones were richer in threonine. On the other hand, a study on the nutritional value of tomatoes failed to confirm a relationship between the type of cultivation and levels of essential amino acids; however, conventional tomatoes had significantly higher content of some non-essential amino acids^20^. The high availability of nitrogen from mineral fertilizers used in conventional farming is the most likely reason for the higher protein content in vegetables grown under this method^5^. Differences in the content of protein and amino acids in favour of conventional vegetables are especially evident in nitrophilic vegetables^21^.

The crude protein content of conventionally grown white and red onion was on average 40% lower than that of organic onions (Tables 1 and 5). The opposite tendency was noted in the case of garlic and leek, i.e. lower (p ≤ 0.05) protein content in organic vegetables. Conventionally grown garlic was the vegetable with the highest crude protein content.

The cultivation model did not significantly affect the content of crude ash in the vegetables, and no substantial differences were noted between types of vegetables (Table 1).

With the exception of yellow onion, significant differences were observed between conventionally and organically grown vegetables in terms of crude fibre content. Fibre content was higher in conventional garlic, red onion and leek (p < 0.001) than in their organic counterparts (Tables 1 and 5). Irrespective of the cultivation system, garlic had the lowest fibre content of all the vegetables. De Souza Araújo et al.^22^ found that organic lettuce and pepper contained significantly more fibre than their conventional counterparts. Similarly, Gontijo et al.^23^ noted a tendency towards higher fibre content in organic lettuce and carrots. Kareem et al.^24^, in a study on sweet potatoes, showed that the use of organic fertilizer instead of mineral fertilizer not only increased the content of crude protein, vitamin A and ether extract, but also reduced the amount of fibre.

### Effect of cultivation system on the content of polyphenolic compounds, vitamin C, and thiocyanates and on catalase activity

*Allium* vegetables are a valuable source of polyphenolic compounds, mainly flavonols (quercetin, kaempferol and their glycosides), as well as phenolic acids^2,25^. Polyphenols are secondary metabolites of plants which are responsible for defence against unfavourable growth conditions, therefore their synthesis may increase as a result of exposure to various stress factors. Polyphenolic compounds have many pharmacological properties, but they especially act as antioxidants^26^. For this reason, their consumption can strengthen antioxidant defence mechanisms and play an important role in the prevention of diseases of free-radical aetiology. The data presented in Table 2 show that yellow onion, irrespective of the cultivation model, contained significantly lower concentrations of total polyphenols (p < 0.05) than the other vegetables. Lenkova et al.^27^, who analysed various *Allium* vegetables in Slovakia, found that they can be ranked as follows in terms of total polyphenol content: chives > red onion > garlic > yellow onion > ramson > white onion. Apart from differences associated with the species or variety, there are many studies indicating that organic crops have higher concentrations of polyphenolic compounds^7,28,29^. The results of our experiment (Tables 2 and 5) showed higher (p < 0.05) total polyphenol content in organically grown vegetables than in conventional ones. Ren et al.^11^ showed that organic cultivation had a beneficial effect on the flavonoid content and antioxidant potential of varieties of red onion. Similar observations, i.e. higher content of flavonoids, were reported for organic Welsh onion^30^. Hallman and Rembiałkowska^31^ also noted higher flavonoid concentrations in organic yellow and red onion varieties. They were also more abundant in anthocyanins. Some studies on *Allium* vegetables, however, including onion^32^ and leek^13^, have not confirmed that organic vegetables are more abundant in polyphenolic compounds than conventionally grown vegetables. Apart from the role of biotic or abiotic stress factors, the increase in synthesis of secondary metabolites in organically grown vegetables may be linked to different fertilization practices^9^. The high levels of easily available mineral nitrogen used in conventional agriculture adversely affect production of polyphenolic substances by plants^33^. On the other hand, a deficiency of nutrients, especially nitrogen, can induce the expression of genes associated with phenylpropanoid and flavonoid synthesis^34^. Faller and Fialho^35^, who analysed various organic and conventional fruits and vegetables, including onion, found that organic onions had lower content of soluble polyphenols than their conventional counterparts. The authors also observed that vegetables modify polyphenol synthesis in response to cultivation conditions to a lesser extent than fruits.

**Table 2:** Content of bioactive compounds and antioxidant activity in *Allium* vegetables depending on the cultivation model (conventional—C; organic—O).

Vegetable	Cultivation model	Garlic	Yellow onion	Red onion	Leek	Statistical analysis		
						VS	CM	VS × CM
Total polyphenols, g(GAE) 100 g^−1^ DW	C	3.05^a^ ± 0.361	0.346^d^ ± 0.056	0.869^c^ ± 0.041	1.47^b^ ± 0.079	**	*	**
	O	4.45^a^ ± 0.325	0.412^d^ ± 0.052	1.17^c^ ± 0.090	2.35^b^ ± 0.068			
	Mean	3.75^a^ ± 0.789	0.379^d^ ± 0.052	1.02^c^ ± 0.017	1.90^b^ ± 0.463			
Vitamin C, mg 100 g^−1^ DW	C	25.11^c^ ± 1.50	74.59^b^ ± 6.01	107.8^a^ ± 9.39	71.36^b^ ± 2.81	**	**	**
	O	51.66^c^ ± 4.03	117.4^b^ ± 10.07	165.1^a^ ± 10.47	114.6^b^ ± 6.38			
	Mean	38.38^c^ ± 13.93	96.01^b^ ± 10.72	136.5^a^ ± 31.04	92.98^b^ ± 22.69			
Thiocyanates, µg 100 g^−1^ DW	C	0.439^b^ ± 0.012	0.391^c^ ± 0.030	0.525^a^ ± 0.026	0.352^d^ ± 0.016	**	**	**
	O	0.471^c^ ± 0.011	0.508^b^ ± 0.012	0.725^a^ ± 0.022	0.524^b^ ± 0.019			
	Mean	0.455^b^ ± 0.020	0.449^b^ ± 0.012	0.625^a^ ± 0.105	0.438^b^ ± 0.090			
Catalase, mg H_2_O_2_ min g^−1^	C	1.77^c^ ± 0.026	1.59^d^ ± 0.011	0.293^b^ ± 0.024	2.16^a^ ± 0.052	**	**	ns
	O	1.13^a^ ± 0.032	0.441^b^ ± 0.033	1.16^a^ ± 0.045	1.15^a^ ± 0.033			
	Mean	1.45^a^ ± 0.330	1.02^b^ ± 0.033	0.727^b^ ± 0.446	1.66^a^ ± 0.520			
FRAP, µmol g^−1^ DW	C	6.38^a^ ± 1.22	2.26^b^ ± 0.444	3.06^b^ ± 0.603	7.06^a^ ± 0.992	**	**	**
	O	7.92^b^ ± 0.953	4.45^d^ ± 0.281	6.04^c^ ± 1.58	10.58^a^ ± 1.03			
	Mean	7.15^b^ ± 1.32	3.36^c^ ± 0.281	4.55^c^ ± 1.32	8.81^a^ ± 2.05			
DPPH^.^, µmol Trolox g^−1^ DW	C	3.81^b^ ± 0.451	2.55^c^ ± 0.121	3.48^b^ ± 0.480	6.05^a^ ± 0.511	**	**	*
	O	4.72^b^ ± 0.571	3.09^c^ ± 0.556	4.79^b^ ± 0.391	7.66^a^ ± 0.433			
	Mean	4.26^b^ ± 0.683	2.82^c^ ± 0.556	4.13^b^ ± 0.800	6.86^a^ ± 0.953			

*VS *vegetable species, *CM *cultivation model (conventional vs. organic), *ns *no statistics.

*Correlation significant at p ≤ 0.05 (one-way analysis).

**Correlation significant at p ≤ 0.01 (one-way analysis).

^a,b^^,c,d^Values with different superscript letters in a row are significantly different at p ≤ 0.05.

In addition to their increased content of phenolic compounds, all of the organically grown *Allium* vegetables had significantly (p < 0.001) higher content of vitamin C, on average over 50% more than their conventional counterparts (Tables 2 and 5). While some researchers report higher content of vitamin C in conventional crops^14,36^, many more studies indicate that organic vegetables contain more vitamin C^29,37–39^. Red onion, both organic and conventional, had the highest content of this vitamin. Golubkina et al.^13^ in all of nine analysed varieties of organic leek, recorded higher levels of vitamin C than in conventionally grown leek. Hallmann and Rembiałkowska^31^ showed that vitamin C content in organic onions was nearly twice as high as in conventional onions. The reason for the higher vitamin C content in organic vegetables is most likely the means of fertilization, specifically the amount of available nitrogen. The dependence of synthesis of carbon and nitrogen compounds on nitrogen availability for plants is explained by the C/N theory, according to which the deficit of readily assimilable nitrogen results in a reduction in photosynthesis and production of nitrogen compounds, while synthesis of carbon-based secondary metabolites increases^40^. This theory can explain the higher levels of vitamin C as well as polyphenolic compounds in organic crops compared to conventional ones.

While the relationship between the means of cultivation and the content of polyphenolic compounds and vitamin C has been the subject of numerous studies, little is known of its effects on the level of thiocyanates. These compounds play a beneficial role as antioxidants and anticarcinogens, but they can also lead to disturbances in thyroid hormone synthesis^41^. Thiocyanates are products of enzymatic hydrolysis of glucosinolates, and their presence is characteristic of cruciferous vegetables. The vegetables analysed in our study had roughly similar content of thiocyanates, but red onion contained significantly more (p ≤ 0.05) than the other vegetables (Table 2). The organic vegetables had higher (p < 0.001) content of thiocyanates than the conventional ones (7% higher for garlic, 30% higher for yellow onion, 38% for red onion, and 49% for leek) (Tables 2 and 5). Research on broccoli has shown that the use of organic fertilizers, especially those rich in sulphur, may increase the synthesis not only of phenolic compounds, but of glucosinolates as well^42^. The content of these substances in organic crops may increase due to their vulnerability to attack by pests or pathogens, as these compounds, like polyphenols, are a defence mechanism^43^.

Biotic or abiotic stress can also induce an increase in the activity of antioxidant enzymes, such as catalase, because exposing plants to stress factors of any type may result in secondary oxidative stress^44^. Catalase is an enzyme with the ability to break down hydrogen peroxide, and together with peroxidase it performs an important function as a regulator of growth and development processes in plants^45^. The results of our experiment showed that catalase activity was higher in conventional vegetables (with the exception of red onion) than in organic vegetables, and the differences were statistically significant (Tables 2 and 5). On the other hand, Sembratowicz and Rusinek-Prystupa^46^ found no effect of the cultivation system on catalase activity in fruits.

Given the wide variation in bioactive substances in vegetables, which can influence their antioxidant activity, the use of a single method to evaluate it is insufficient. For this reason we assessed the ferric antioxidant potential (FRAP) of the vegetables as well as their capacity to reduce the synthetic DPPH^⋅^ radical. The results of both tests indicated that yellow onion had the weakest (p ≤ 0.05) antioxidant properties among all the vegetables tested (Table 2). This was correlated with the lowest content of polyphenolic compounds. A study evaluating the activity of various *Allium* vegetables^27^ found that yellow onion placed before garlic and white onion. All organic vegetables in our study (p ≤ 0.05) proved superior to conventional ones in terms of ferric-reducing (FRAP) and DPPH^⋅^ radical-scavenging capacity (Tables 2 and 5). The higher antioxidant activity of organic crops has been confirmed in experiments on various vegetables^10,12^, including those of the genus *Allium*^11^. In our study, the greatest differences between cultivation models for antioxidant activity as determined by the FRAP method were shown in red and yellow onion, in which it was about twice as high in organic vegetables as in conventional ones (Table 2). The correlation coefficients for the FRAP vs DPPH^⋅^ methods calculated for each vegetable indicate a fairly strong, statistically significant correlation between these methods (Table 3). As the FRAP and DPPH^⋅^ assays are based on the same mechanism, i.e. the transfer of a single electron from the antioxidant to the oxidant, a strong relationship between the methods can be expected. This is confirmed by the results of other studies analysing the antioxidant activity of fruits^47^ and vegetables^48^.

**Table 3:** Pearson correlation coefficients between the content of DPPH^⋅^ and that of total polyphenols, vitamin C, and FRAP.

	Vegetable	Vitamin C	FRAP	DPPH^.^
Total polyphenols	Garlic	0.862**	0.509*	0.642**
	Yellow onion	0.551*	0.596**	0.329
	Red onion	0.884**	0.750**	0.769**
	Leek	0.958**	0.815**	0.882**
Vitamin C	Garlic		0.576**	0.661**
	Yellow onion		0.920**	0.418*
	Red onion		0.782**	0.842**
	Leek		0.861**	0.851**
FRAP	Garlic			0.597**
	Yellow onion			0.538*
	Red onion			0.635**
	Leek			0.763*

*Correlation significant at p ≤ 0.01 (one-way analysis).

**Correlation significant at p ≤ 0.001 (one-way analysis).

For most of the vegetables (except for yellow onion) there was a strong positive correlation (r = 0.509–0.815, p ≤ 0.001) between the content of polyphenolic compounds and FRAP (Table 3). DPPH^⋅^ radical-scavenging ability was also strongly correlated with the content of polyphenols (r = 0.642–0.882, p ≤ 0.001). Only in the case of yellow onion was the relationship slightly weaker and statistically non-significant (r = 0.329, p > 0.05). Various experiments have shown that polyphenolic compounds have an important role in the antioxidant activity of *Allium* vegetables^27,49^. The data presented in Table 3 show that the antioxidant activity of the vegetables was also strongly correlated with the content of vitamin C. The slightly higher correlation coefficients between the FRAP value and vitamin C than between FRAP and polyphenolic content in the vegetables may indicate that vitamin C had a greater influence on their capacity to reduce Fe^+3^ ions than did polyphenolic compounds. There are reports indicating that the antioxidant activity of vegetables is more dependent on other chemical ingredients than vitamin C^50^. In the case of *Allium* vegetables, apart from polyphenols and vitamin C, their antioxidant activity is also determined by the presence of sulphur compounds^51^.

### Mineral content

Apart from the content of antioxidants, such as polyphenols and vitamin C, the vegetables were also analysed for content of micronutrients (copper, iron, manganese and zinc) functioning as coenzymes of antioxidant enzymes^52^. It should be borne in mind that these elements can be introduced to the environment from anthropogenic sources (e.g. transport and industry), and if they accumulate in large amounts they can be toxic for plants and pose a threat to consumer. The results indicate a marked tendency (p ≤ 0.05) of higher levels of these minerals in organic vegetables relative to conventionally grown vegetables. Only in the case of leek was the content of copper higher (p = 0.005) in the conventional system (Tables 4 and 5). The most pronounced differences in the content of iron, copper and zinc were noted in yellow onion, with 71%, 79% and 60% higher content in the organic model than in the conventional model. Ilić et al.^53^ reported that with the exception of copper, organically grown green onion had higher content of microelements and much higher levels of macroelements than conventional green onion. Głodowska and Krawczyk^54^ analysed various conventionally and organically grown vegetables (leek, beetroot, celeriac, parsley, potato and onion) from Poland. They found that conventional parsley root and beetroot had higher content of copper than their organic counterparts, while the reverse relationship was found for potato. Iron content was higher in conventionally grown vegetables, in contrast with zinc content, which was higher in organic vegetables. Domagała-Świątkiewicz and Gąstoł^55^, who analysed juices from organic and conventionally grown beetroot, carrot and celeriac, found that organic juices had higher content of calcium and lower content of cadmium than their conventional counterparts. However, there was no general trend pointing to the advantage of one model over the other in terms of the content of other minerals.

**Table 4:** Content of minerals (mg 100 g^−1^ DW) in *Allium* vegetables depending on the cultivation model (conventional—C; organic—O).

Vegetable	Cultivation model	Garlic	Yellow onion	Red onion	Leek	VS	CM	VS × CM
Magnesium	C	23.98^a^ ± 2.11	6.79^c^ ± 0.33	6.92^c^ ± 0.53	10.81^b^ ± 0.55	**	ns	**
	O	31.88^a^ ± 2.93	8.54^c^ ± 0.89	7.25^c^ ± 0.90	12.30^b^ ± 1.12			
	Mean	27.93^a^ ± 4.76	7.67^c^ ± 0.891	7.08^c^ ± 0.74	11.57^b^ ± 1.16			
Calcium	C	90.77^a^ ± 8.03	23.07^c^ ± 2.26	22.77^c^ ± 1.33	48.50^b^ ± 3.38	**	ns	**
	O	114.5^a^ ± 9.16	30.39^c^ ± 2.44	28.84^c^ ± 3.03	55.55^b^ ± 4.83			
	Mean	102.6^a^ ± 14.78	26.73^c`^ ± 2.44	25.80^c^ ± 3.86	52.02^b^ ± 5.43			
Iron	C	6.12^a^ ± 0.695	1.17^c^ ± 0.062	3.55^b^ ± 0.201	1.28^c^ ± 0.068	**	ns	**
	O	8.18^a^ ± 0.526	2.00^c^ ± 0.041	4.58^b^ ± 0.248	1.44^d^ ± 0.067			
	Mean	7.15^a^ ± 1.21	1.59^c^ ± 0.041	4.06^b^ ± 0.581	1.36^c^ ± 0.11			
Zinc	C	1.22^a^ ± 0.062	0.433^c^ ± 0.032	1.11^b^ ± 0.061	0.374^d^ ± 0.015	**	*	*
	O	1.51^a^ ± 0.115	0.695^b^ ± 0.051	1.46^a^ ± 0.032	0.564^c^ ± 0.022			
	Mean	1.36^a^ ± 0.175	0.564^b^ ± 0.051	1.29^a^ ± 0.186	0.469^b^ ± 0.099			
Copper	C	0.136^a^ ± 0.012	0.141^a^ ± 0.013	0.024^b^ ± 0.007	0.031^b^ ± 0.005	**	*	**
	O	0.188^b^ ± 0.023	0.252^a^ ± 0.022	0.033^c^ ± 0.009	0.022^c^ ± 0.007			
	Mean	0.162^b^ ± 0.032	0.197^a^ ± 0.022	0.028^c^ ± 0.009	0.027^c^ ± 0.007			
Manganese	C	0.138^b^ ± 0.012	0.139^b^ ± 0.011	0.166^a^ ± 0.014	0.168^a^ ± 0.013	**	**	ns
	O	0.173^b^ ± 0.011	0.175^b^ ± 0.018	0.219^a^ ± 0.012	0.210^a^ ± 0.015			
	Mean	0.155^b^ ± 0.021	0.157^b^ ± 0.018	0.193^a^ ± 0.030	0.189^a^ ± 0.026			

*VS* vegetable species, *CM *cultivation model (conventional vs. organic).

*Correlation significant at p ≤ 0.05 (one-way analysis).

**Correlation significant at p ≤ 0.01 (one-way analysis).

^a,b^^,c,d^Values with different superscript letters in a row are significantly different at p ≤ 0.05.

**Table 5:** Results of one-way analysis of variance (ANOVA) used to determine the effect of the cultivation model (conventional vs. organic) on individual *Allium* vegetables and on individual parameters included in the sample.

Vegetables	Garlic	Yellow onion	Red onion	Leek
Dry matter	< 0.001	0.247	0.627	< 0.001
Crude protein	< 0.001	< 0.001	< 0.001	< 0.001
Crude ash	0.012	< 0.001	< 0.001	0.576
Crude fibre	< 0.001	0.850	< 0.001	0.003
Total polyphenols	< 0.001	0.013	< 0.001	< 0.001
Vitamin C	< 0.001	< 0.001	< 0.001	< 0.001
Thiocyanates	< 0.001	< 0.001	< 0.001	< 0.001
CAT	< 0.001	< 0.001	< 0.001	< 0.001
FRAP	0.006	< 0.001	< 0.001	< 0.001
DPPH^⋅^	0.001	0.008	< 0.001	< 0.001
Magnesium	< 0.001	< 0.001	0.324	0.001
Calcium	< 0.001	< 0.001	< 0.001	0.001
Iron	< 0.001	< 0.001	< 0.001	< 0.001
Zinc	< 0.001	< 0.001	< 0.001	< 0.001
Copper	< 0.001	< 0.001	0.027	0.005
Manganese	< 0.001	< 0.001	< 0.001	< 0.001

The higher contents of various micronutrients and macronutrients in organic vegetables may be explained by the effect of fertilization. Organic fertilizers used in organic farming are conducive to the development of arbuscular mycorrhizal fungi and other beneficial soil organisms (microbes and earthworms)^56^. Their activity in decaying organic matter leads to the formation of vermicompost, a source of both macroelements and microelements which are readily available for plants^57^.

The macronutrients analysed in the *Allium* vegetables were calcium and magnesium (Table 4). Organic vegetables were shown to have higher content of these minerals than conventional ones, but in the case of red onion (Mg) the differences were not statistically significant (Tables 4 and 5). A survey of research on the nutritional quality of organic and conventional crops^18^ found that the former had higher concentrations of iron, magnesium, and phosphorus. A study comparing organic and conventional potatoes showed that organic production resulted in lower levels of sodium and iron but higher concentrations of magnesium and copper^58^. In our study, organic garlic was the vegetable with the highest magnesium content, which was more than three times that of the other organic vegetables (Table 4). It should be noted that conventional garlic contained more magnesium than the other *Allium* vegetables from either cultivation model (except for organic garlic). Higher magnesium content in juices from conventional beetroot and carrot was shown in the previously cited study by Domagała-Świątkiewicz and Gąstoł^55^. On the other hand, Głodowska and Krawczyk^59^ noted a tendency towards higher Ca and Mg contents in vegetables grown organically, although they were not statistically significant in all of them.

It should be noted, however, that besides the cultivation model, the content of minerals in crop plants may be influenced by numerous other factors, such as soil type, climate conditions, stage of maturity, or exposure to pollution^54^. In addition, accumulation of individual mineral elements in vegetables is species-specific. In our study, irrespective of the differences associated with the cultivation model, the highest content of macronutrients Ca and Mg and micronutrients Fe and Zn was noted in garlic. Among the vegetables analysed, the highest copper content was found in yellow onion and the highest manganese content in red onion and leek (Table 4).

The results of the study confirm the influence of the cultivation method on the nutritional value and antioxidant capacity of *Allium* vegetables. The content of crude protein, crude fibre, and dry matter in organic garlic was lower than in conventional garlic. In contrast, vegetables from organic cultivation had much higher content of minerals, as well as compounds with antioxidant properties.

The species of *Allium* vegetable influenced the content of bioactive substances. Among the vegetables tested, garlic had significantly higher content of dry matter, total protein and minerals, i.e. magnesium, calcium, iron and zinc. In the case of compounds with potential antioxidant activity, garlic was the most abundant in total polyphenols, and red onion in vitamin C. Leek showed the highest CAT activity, as well as FRAP and DPPH^.^ values.

## Materials and methods

### Samples

The research material consisted of organically and conventionally grown vegetables of the same cultivars belonging to the genus *Allium*: garlic (*Allium sativum* L.); leek (*Allium ampeloprasum* L*.*); and yellow and red onion (*Allium cepa* L.). The collection of plant material and all methods were performed according to the relevant guidelines and regulations. The vegetables were obtained from two certified organic farms and two conventional farms located in Tarnogród commune. The plants were grown in similar climate and soil conditions. Fertilization and pest control on the organic and conventional farms were in accordance with applicable principles.

For each vegetable grown on the two organic and two conventional farms, ten samples comprising three specimens of the vegetable were analysed. Each analysis was performed in duplicate. In total 320 samples were analysed (4 vegetables × 2 cultivation models × 2 farms × 10 samples × 2 replicates).

The edible parts of freshly washed vegetables (without skins in the case of garlic and onion) were homogenized in a BUCHI B-400 mixer with ceramic knives. In the case of leek, the entire edible part was used, i.e. the white part and the green part. The homogenized sample was divided into three portions. The first portion was weighed, dried, and analysed for the content of basic nutrients (dry matter, crude protein, crude ash, and crude fibre). The second portion was placed in plastic tubes and deep-frozen at −80 °C until analysis for the content bioactive substances. The third portion was used for determination of mineral content.

### Chemical analysis

The homogenized vegetables were assayed for content of dry matter, ash, crude fibre and crude protein according to AOAC methods^60^.

Polyphenolic compounds were extracted as described by Bakowska-Barczak and Kolodziejczyk and Czech et al.^61,62^. Their total content was determined spectrophotometrically by the Folin-Ciocalteu method^63^. The absorbance of samples was measured at 760 nm after two hours of incubation in the dark. Total polyphenol content was calculated from a standard curve (y = 0.0059x + 0.0401, *R*^2^ = 0.9962) prepared for a known concentration of gallic acid and expressed as grams of gallic acid equivalent (GAE)/100 g DW of sample.

Vitamin C was determined spectrophotometrically following the Folin-Ciocalteu reagent method^64^. The absorbance of samples was measured after 10 min of incubation at room temperature at 760 nm. The content of vitamin C was calculated from a standard curve (y = 0.0208x − 0.0035; R^2^ = 0.9998) with a known concentration of vitamin C.

For analysis of thiocyanates, 5 g of homogenized sample was weighed out. One gram of sample was placed in a stoppered 10 ml test tube, 9 ml of 5% trichloroacetic acid (TCA) was added, and the tube was shaken for 10 min. The samples were centrifuged at 3000 rpm for 10 min and filtered through filter paper. After thorough mixing, 2 ml of filtrate was placed in each of two test tubes. For the blank sample, 2 ml of distilled water was added to one of the tubes, and 2 ml of ferric nitrate was added to the other. From that point the samples were kept in the dark. A reagent blank was also prepared from 2 ml of water and 2 ml of ferric nitrate. Then the absorbance of the samples and blanks was measured against distilled water at 470 nm, no later than 5 min after the addition of ferric nitrate^65^. The concentration of thiocyanates in the sample was read from a standard curve (y = 7.1529x + 0.0237; R^2^ = 0.9991), subtracting the value of the blank samples (blank and reagent blank) from the absorbance. The result was expressed per 100 g of product.

Catalase (CAT) activity was determined by the manganometric method^66^, which consists in titration with KMnO_4_ in an acidic environment of the remaining undecomposed H_2_O_2_. CAT activity was expressed as milligrams of degraded H_2_O_2_ per gram of sample per min.

DPPH^⋅^ free radical scavenging activity was measured according to Abeysekera et al.^67^. Due to the reduction of the radical by the antioxidants present in the sample, there is a decrease in absorbance at 517 nm, which is measured after 15 minutes of incubation in the dark. The exact DPPH^⋅^ concentration remaining in the solution was calculated from a standard curve (y = 0.0011x − 0.0747; R^2^ = 0.988) prepared for a known concentration of Trolox.

The FRAP (ferric-reducing antioxidant power) assay was performed as described in detail elsewhere^68^. Antioxidants in the sample reduce ferric-tripyridyl triazine complex to the blue product with maximum absorption at 593 nm. The change in absorbance over 4 min is proportional to the FRAP value of the antioxidants in the sample. Aqueous solutions of ferrous ions (iron (II) sulphate (FeSO_4_⋅7H_2_O) were used as standard solutions.

All spectrophotometric analyses were performed using the Unicam 5625 UV/VIS (United Kingdom) spectrophotometer.

To determine the content of minerals, homogenized samples were subjected to wet ashing (HNO_3_, Sigma Aldrich). Following mineralization and cooling to room temperature, the sample was made up to volume with demineralized water (ELGA Pure Lab Classic) in a 50 ml flask.

Mineral elements were determined by ICP-OES (inductively coupled plasma optical emission spectrometry) with a Thermo Scientific iCAP Series 6500 spectrometer equipped with a charge injection device (CID) detector. The spectrometer was controlled by PC-based iTEVA software. The following multi-element stock solutions (Inorganic Ventures) were used as standards:

(A) Standard 1—46: 63Cu, 57Fe, 24Mg, 40Ca in 5% HNO_3_—1000 mg kg^−1^.

(B) Standard 2—47: 55Mn, 79Se, 66Zn in 10% HNO_3_—100 mg kg^−1^.

All concentrations obtained in the study are given in mg kg^−1^ dry weight (DW).

### Statistical analysis

The digital data obtained were subjected to statistical analysis, in which mean values and standard errors of the means were determined using Statistica ver. 10 software. The normality of data distribution was tested using Shapiro-Wilk test. Two-factorial analysis of a model including the vegetable species (VS) and the cultivation model (conventional vs. organic—CM) as well as the interaction between both factors was conducted (VS × CM). The significance of differences between means was determined using Duncan’s test at significance levels of p ≤ 0.05 and p ≤ 0.01. Pearson correlation coefficients between DPPH^⋅^ and FRAP values, total polyphenol content, and vitamin C content were estimated for the *Allium* vegetables.

## Data Availability

The datasets generated and analysed during the study are available from the corresponding author on reasonable request.
